# Risk Factors for Mucosal Redness in the Duodenal Bulb as Detected via Linked Color Imaging

**DOI:** 10.3390/diagnostics14050508

**Published:** 2024-02-28

**Authors:** Tsutomu Takeda, Daiki Abe, Daisuke Asaoka, Tomoyo Iwano, Momoko Yamamoto, Ryota Uchida, Hisanori Utsunomiya, Shotaro Oki, Nobuyuki Suzuki, Atsushi Ikeda, Yoichi Akazawa, Kumiko Ueda, Hiroya Ueyama, Mariko Hojo, Shuko Nojiri, Akihito Nagahara

**Affiliations:** 1 Department of Gastroenterology, Juntendo University School of Medicine, Tokyo 113-8421, Japan; d-abe@juntendo.ac.jp (D.A.); t-iwano@juntendo.ac.jp (T.I.); mm-yamamoto@juntendo.ac.jp (M.Y.); r-uchida@juntendo.ac.jp (R.U.); h-utsunomiya@juntendo.ac.jp (H.U.); s-oki@juntendo.ac.jp (S.O.); nb-suzuki@juntendo.ac.jp (N.S.); at-ikeda@juntendo.ac.jp (A.I.); yakazawa@juntendo.ac.jp (Y.A.); ktamaki@juntendo.ac.jp (K.U.); psyro@juntendo.ac.jp (H.U.); mhojo@juntendo.ac.jp (M.H.); nagahara@juntendo.ac.jp (A.N.); 2 Department of Gastroenterology, Juntendo Tokyo Koto Geriatric Medical Center, Tokyo 136-0075, Japan; daisuke@juntendo.ac.jp; 3 Department of Medical Technology Innovation Center, Juntendo University School of Medicine, Tokyo 113-8421, Japan; s-nojiri@juntendo.ac.jp

**Keywords:** abdominal symptoms, duodenum, linked color imaging, mucosal redness

## Abstract

Linked color imaging (LCI) for image-enhanced endoscopy (IEE) highlights mucosal color differences. We investigated risk factors associated with mucosal redness of the duodenal bulb using LCI. Consecutive patients were retrospectively selected after their duodenal bulbs were observed via LCI. A symptom questionnaire (Izumo scale) was completed. The LCI of the duodenal bulb was subjectively evaluated on whether redness was present and objectively evaluated based on L* a* b* color values. The clinical characteristics of the 302 study participants were: male/female, 120/182; mean age, 70.9 years. Twenty-one cases (7.0%) were in the redness (+) group. After multiple regression analysis, independent predictors for the red component (a*) of the duodenal bulb using LCI were: age (β = −0.154, *p* < 0.01), female (β = −0.129, *p* < 0.05), body mass index (BMI; β = −0.136, *p* < 0.05), *Helicobacter pylori* eradication (β = 0.137, *p* < 0.05), endoscopic gastric mucosal atrophy score (EGAS; β = −0.149, *p* < 0.05), and constipation-related quality of life (QOL) (β = −0.122, *p* < 0.05) scores. Lower age, lower BMI, lower EGAS, a constipation-related QOL score, post-*H. pylori* eradication, and being male were associated with mucosal redness in the duodenal bulb with IEE using LCI.

## 1. Introduction

Despite the presence of abdominal symptoms, organic disease is not evident in functional gastrointestinal diseases (FGIDs). Comorbidities exist with FGIDs, including functional constipation (FC), functional dyspepsia (FD), gastroesophageal reflux disease (GERD), and irritable bowel syndrome (IBS). These comorbidities show an extremely high incidence of 40.3% as suggested by a multi-national study involving an Internet survey [1]. Functional constipation is the most common digestive disorder, with a rising incidence evident with increasing age [2]. In Japan, the common occurrence of constipation was reported by 28.4% of respondents in an Internet survey [3]. The consequence of constipation in patients is decreased quality of life (QOL) [4]. Additionally, FD contributes to economic loss due to impaired QOL since it is a chronic functional disease that causes pain or discomfort centered in the upper abdomen [5]. Therefore, combatting FGIDs has become important.

Infection by *Helicobacter pylori* can lead to dyspeptic symptoms. Microscopic duodenitis in *H. pylori*-infected patients may lead to, and aggravate, symptoms of FD [6]. In recent years, a new concept of the relationship between the duodenum and FD has attracted attention [7,8]. The infiltration of eosinophils, lymphocytes or mast cells in the duodenal mucosa indicates duodenal immune activation and low-grade inflammation in FD [9,10,11,12]. Although the duodenum plays an important role in the pathogenesis of FD, endoscopic assessment of the duodenum is less frequent than that of the stomach.

The endoscopic diagnosis of the duodenum has been reported mainly for celiac disease through the evaluation of duodenal villous atrophy [13,14,15]. Subsequently, image-enhanced endoscopies (IEEs), such as narrow-band imaging and I-scan, have been developed, with duodenal villous morphology subsequently evaluated using IEEs [16,17]. As a new system of IEE, linked color imaging (LCI) elevates color tone and boosts visibility. Color separation in the red regions of the gastric mucosa can be improved by LCI. As a consequence, red lesions are easier to identify with this system. Diffuse redness, indicating *H. pylori*-positive findings, is emphasized in LCI [18,19]. In this regard, we previously reported on the improved visibility of diffuse redness in LCI compared to white light imaging (WLI) [20]. However, the significance of mucosal redness in the duodenum that resembled gastric diffuse redness as detected with LCI was unclear. In this study on LCI, we characterized the risk factors that influenced mucosal redness in the duodenal bulb.

## 2. Materials and Methods

### 2.1. Study Design

We undertook this single-center study within a university hospital (Juntendo Tokyo Koto Geriatric Medical Center). This was a retrospective and cross-sectional study that was performed from April 2017 to March 2020. We elucidated the relationship between abdominal symptoms and mucosal redness in the duodenal bulb as observed via endoscopy with LCI. We used EG-L600WR7, EG-L590WR, or EG-L600ZW7 (Fujifilm, Tokyo, Japan) endoscope systems, LASEREO7000 VP-7000 or AdvanciaHD VP-4450HD (Fujifilm, Structure Emphasis: B6, Color Emphasis: C1) video processors, and LASEREO7000 LL-7000 or LL-4450 (Fujifilm Corporation) light sources. An esophagogastroduodenoscopy (EGD) was performed in the morning on patients who had fasted for the previous 12 h. A symptom questionnaire concerning quality of life (QOL; Izumo scale) that related to abdominal symptoms was self-administered before an endoscopy procedure on the day of examination. After an endoscopic examination of the esophagus, the stomach was then similarly examined. The duodenum was examined before the stomach; in the duodenum, images were recorded in WLI and LCI close to the anterior wall of the duodenal bulb using air insufflation. Linked color imaging of redness in the duodenal bulb was subjectively assessed by endoscopists and objectively evaluated based on L* a* b* color values in a CIELAB color space system. Multiple regression analysis was undertaken to retrospectively examine the relationship between mucosal redness in the duodenal bulb using LCI and each factor, including abdominal symptoms.

### 2.2. Inclusion Criteria

Consecutive patients in the stated period were included in the study based on obtaining the following complete information from personal health records:

(1) Personal characteristics (body mass index [BMI], age, and gender);
(2) History of alcohol and tobacco use;
(3) Status of *H. pylori* infection (positive, negative, or negative after eradication);
(4) Use of a potassium competitive acid blocker (PCAB), proton pump inhibitor (PPI), antiplatelet, or non-steroidal anti-inflammatory drugs (NSAIDs);
(5) EGD results (reflux esophagitis (RE_), endoscopic gastric mucosal atrophy score (EGAS), and LCI images of the duodenal bulb;
(6) QOL (Izumo scale) related to abdominal symptoms.

### 2.3. Exclusion Criteria

We excluded patients who showed the following in their medical history: inflammatory bowel disease, erosive duodenitis, gastrointestinal surgery, advanced gastrointestinal cancer, an active duodenal or gastric ulcer, celiac disease, deformity because of duodenal scars, leukemia, multiple myeloma, mental illness, or a malignant lymphoma. The stomach was sprayed with l-menthol before observation of the duodenum. In addition, patients were excluded if the duodenal bulb could not be clearly observed due to bubbles, mucus, bile, and halation. Patients were also excluded if they had a history of cerebrovascular, acute gastrointestinal, coronary, renal, respiratory, or hepatic events.

### 2.4. Assessments of Clinical Parameters

Calculations of BMI were based on the division of body weight by body height in m^2^ (kg/m^2^). A history of alcohol was specified as daily consumption of 12 g or more of pure alcohol. A history of tobacco use was defined as the current smoking of ≥1 cigarette daily. Patients were considered to have an *H. pylori* infection if a 13C-urea breath test was positive and/or they had specific antibodies in their serum. Eradication treatment was deemed successful if a negative result for *H. pylori* infection was obtained 4 to 8 weeks after treatment ending. A PPI/PCAB user was deemed to be someone who used any of five types of PPI/PCAB daily (lansoprazole, rabeprazole, esomeprazole, vonoprazan, or omeprazole) for more than 2 months. Users of antiplatelet drugs (clopidogrel, ticlopidine, prasugrel, trapidil, aspirin, dipyridamole, beraprost, cilostazol, nicorandil, limaprost alfadex, sarpogrelate, or icosapent ethyl) or NSAIDs (diclofenac sodium, felbinac, indomethacin, bromfenac, etodolac, lornoxicam, meloxicam, ibuprofen, naproxen, ketoprofen, loxoprofen, celecoxib, or aspirin) were defined as those who had used them daily. Patients who took a normal dose of a PPI/PCAB, antiplatelet drug, or NSAID were considered to use such treatments. As an exception, low doses of aspirin were also included.

### 2.5. EGD Findings

For EGD findings, patients were characterized with RE of grade A, B, C, or D based on a partially revised Los Angeles (LA) classification system [21]. Endoscopic gastric mucosal atrophy was classified as C-0 (normal), C-1, C-2, C-3, O-1, O-2, or O-3 [22], in addition to the position of the endoscopic atrophic border, according to the Kimura–Takemoto classification system. EGAS values were assigned according to atrophy: 0 = C-0 type, 1 = C-1 type, 2 = C-2 type, 3 = C-3 type, 4 = O-1 type, 5 = O-2 type, or 6 = O-3 type. Each group of patients had a mean EGAS calculated.

### 2.6. Questionnaire for Abdominal Symptom-Related QOL

Abdominal symptom-related QOL was quantitatively measured using the Izumo scale [23,24,25]. This consisted of a questionnaire that was self-reported with 15 items within five domains: reflux, fullness, upper abdominal pain, diarrhea, and constipation. Scores were from 0 to 5 for each item on a Likert scale as shown: 0 = not bothered, 1 = not so bothered, 2 = slightly bothered, 3 = bothered, 4 = strongly bothered, and 5 = intolerably bothered. Each domain was made up of three items. The severity of symptoms in each domain was scored between 0 and 15 points, with severity increasing with the number of points. Each question fell into one of the five domains: the reflux domain covered questions 1, 2, and 3; the upper abdominal pain domain encompassed questions 4, 5, and 6; the fullness domain covered questions 7, 8, and 9; the constipation domain covered questions 10, 11, and 12; and the diarrhea domain covered questions 13, 14, and 15. Impairments in domain-specific QOL, according to the Izumo scale, were categorized between 0 (no impairment) and 15; these were internally consistent and correlated well with the Gastrointestinal Symptom Rating Scale. Upper abdominal pain-, reflux-, constipation-, fullness-, and diarrhea-related QOL scores for each patient were assigned according to the sum of scores for three questions per domain (reflux, upper abdominal pain, fullness, constipation, and diarrhea).

### 2.7. Subjective Evaluations of the Mucosal Redness of the Duodenal Bulb

Two experts (gastroenterologists who were board-certified and members of the Japan Gastroenterological Endoscopy Society; TT, DA2) assessed the endoscopic findings and classified them into redness (+) and redness (−) groups. Patients in the redness (+) group were characterized with findings of diffuse redness, in an LCI observation, under the anterior wall of the duodenal bulb with air insufflation. No redness or partial redness characterized the findings of patients in the redness (−) group. Assessments were made by blindly examining the clinical information of the cases; a decision was made by consensus in the case of disagreement. Each assessor was exposed to independent and random images displayed against a black background (10.3 × 12.9 cm) on a screen (Microsoft Office PowerPoint 2019, Microsoft Inc., Redmond, WA, USA).

### 2.8. Objective Analysis of the Mucosal Color of the Duodenal Bulb with LCI

As described previously, images were evaluated using L* a* b* (L* = light/dark; a* = red/green; b* = yellow/blue) color scores in a Commission Internationale de l’Éclairage (CIE) LAB color space system [26] and Adobe^®^ Photoshop CC 2019 [27]. The anterior wall of the duodenal bulb was used to delineate a region of interest (ROI; 400 × 400 pixels). In the ROI, color values (*L*, *a*, *b*), in addition to their average, were determined using a histogram panel. *L*, *a*, and *b* defined color scores in Photoshop (Lab color unit). The *L*, *a*, and *b* color values were changed to L* a* b* color values in CIELAB according to: L* = *L*/256 × 100, a* = *a* − 128, and b* = *b* − 128 [28,29].

### 2.9. Statistical Analyses

A Wilcoxon rank sum test was used to assess statistically significant differences in L* a* b* color values between WLI and LCI. A Mann–Whitney *U* test was used to analyze differences between the redness (+) and (−) groups in L* a* b* color values. Correlations between the red component (a*) of the duodenal bulb with LCI and various clinical parameters (gender, age, history of alcohol and tobacco use, BMI, reflux esophagitis, *H. pylori* infection status—positive or eradicated, atrophic gastritis, antiplatelet drug use, NSAID use, PPI/PCAB use, and reflux-, upper abdominal pain-, fullness-, constipation-, and diarrhea-related QOL scores) were calculated using Spearman’s correlation coefficient. For multiple regression analysis, the red component (a*) of the duodenal bulb with LCI was employed as a dependent variable. Age, gender, BMI, atrophic gastritis, reflux esophagitis, *H. pylori* infection (positive or eradicated), antiplatelet drug use, NSAID use, PPI/PCAB use, a history of tobacco and alcohol use, and reflux-, upper abdominal pain-, fullness-, constipation-, and diarrhea-related QOL scores were deemed independent variables. Risk factors for the red component (a*) of the duodenal bulb with LCI underwent multiple regression analyses using a stepwise method. Multicollinearity was based on a variance inflation factor of ≥10. SPSS for Windows, version 28.0 (SPSS, Inc., Chicago, IL, USA) was used in the statistical analyses. A statistically significant difference was considered as *p* < 0.05.

## 3. Results

### 3.1. Patients’ Clinical Characteristics

**Figure 1:**
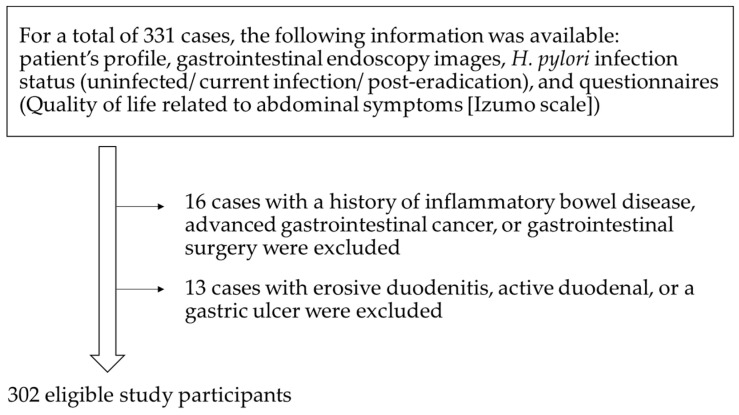
Flow chart of study participants.

For our study, 331 cases met our inclusion criteria. Of these, 302 cases were enrolled in this study (Figure 1). Table 1 shows the clinical characteristics of the study participants. The participants showed a mean age of 70.9 years (range: 27–92). Of the 302 participants, 182 were female and 120 were male. The participants showed a mean BMI of 22.8. Some participants (7.9%) had erosive reflux esophagitis. 76 participants had an *H. pylori* infection (positive), 186 negative, and 40 were classed as post-eradication. In total, 207 had atrophic gastritis (closed: 89, open: 118) and 95 patients did not. Of these, 111 were taking a PCAB or PPI. Antiplatelet drugs were used by 59 patients. Nineteen patients used alcohol and six used tobacco.

### 3.2. Abdominal Symptom Questionnaires

Table 2 shows the results of the abdominal symptom questionnaires. The median (range) values of each symptom questionnaire QOL score were reflux-related, 0 (0–11); upper abdominal pain-related, 0 (0–14); fullness-related, 0 (0–12); constipation-related, 1 (0–10); and diarrhea-related, 0 (0–15).

### 3.3. Subjective Evaluations and Objective Analyses of the Mucosal Color of the Duodenal Bulb

**Figure 2:**
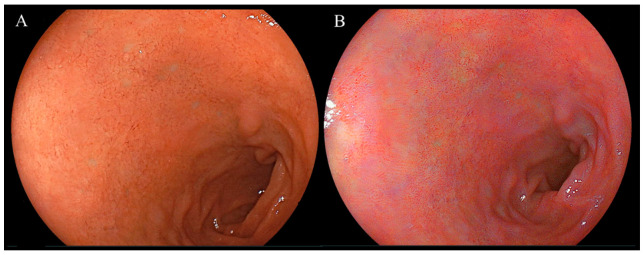
Representative endoscopic images of the duodenal mucosa of bulbs showing red mucosa using white light imaging and linked color imaging. (**A**) White light imaging (WLI). Duodenal mucosa of bulbs. (**B**) Linked color imaging (LCI). The duodenal mucosa of bulbs was accentuated in a red color using LCI.

**Figure 3:**
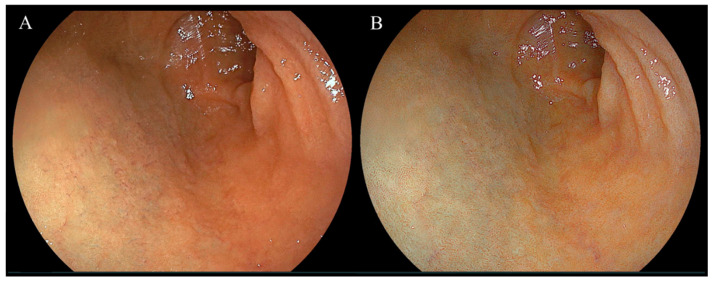
Representative endoscopic images of the duodenal mucosa of bulbs not showing red mucosa using white light imaging and linked color imaging. (**A**) White light imaging (WLI). Duodenal mucosa of bulbs. (**B**) Linked color imaging (LCI). The duodenal mucosa of bulbs was not visible in a red color using LCI.

**Figure 4:**
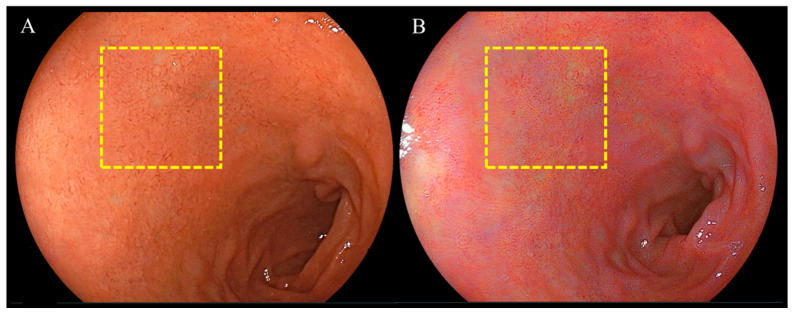
Representative endoscopic images using (**A**) white light imaging or (**B**) linked color imaging in the duodenal mucosa of bulbs. Dashed yellow lines demarcate regions of interest (ROIs; 400 × 400 pixels). The ROIs in the anterior wall of the duodenal bulb were localized in the same positions for each specific lesion.

Endoscopic images taken in the duodenal mucosa of bulbs, with or without red mucosa, are shown in Figure 2 and Figure 3, respectively. Approximately 21 out of 302 (7.0%) cases were in the redness (+) group and the concordance rate between the two assessors was 86.4% (261/302). Figure 4 presents endoscopic images after the use of WLI and LCI with ROIs. An analysis of L* a* b* color values for the duodenal bulb was carried out (Table 3). White light imaging and LCI showed significant differences between the L* a* b* color values. The red component (a*) of the duodenal bulb with LCI was significantly higher than that using WLI in the redness (+) group but was found to be significantly lower in the redness (−) group. Comparing the L* a* b* color values between redness (+) and (−) groups, the a* values were significantly higher in the redness (+) group for both WLI and LCI (*p* < 0.001). However, a difference was not observed in b* values with LCI (WLI: *p* = 0.021, LCI: *p* = 0.338). Subjective evaluation showed that the redness (+) group consisted of 7% of 302 cases. Objective evaluation showed that the red component (a*) of the duodenal bulb with LCI was significantly higher than WLI in the redness (+) group.

### 3.4. Correlation between the Red Component (a*) of the Duodenal Bulb with LCI and Clinical Parameters

Table 4 shows Spearman’s correlation coefficients. For the red component (a*) of the duodenal bulb with LCI, correlation coefficients that were statistically significant (*p* < 0.05) were identified for age (Rho = −0.223, *p* < 0.001), *H. pylori* eradication (Rho = 0.149, *p* = 0.010), EGAS (Rho = −0.149, *p* = 0.010), PPI/PCAB users (Rho = −0.138, *p* = 0.017), and the upper abdominal pain-related QOL score (Rho = 0.118, *p* = 0.041).

### 3.5. Multiple Regression Analysis

Table 5 shows the findings of the multiple regression analysis. Statistically significant independent predictors for the red component (a*) of the duodenal bulb with LCI were age (standardized partial regression coefficient [β] = −0.154, *p* = 0.009), female (β = −0.129, *p* = 0.024), BMI (β = −0.136, *p* = 0.016), *H. pylori* eradication (β = 0.137, *p* = 0.015), EGAS (β = −0.149, *p* = 0.013), and the constipation-related QOL score (β = −0.122, *p* = 0.027). Thus, a lower age, being male, lower BMI, post-*H. pylori* eradication, lower EGAS, and lower constipation-related QOL score were associated with mucosal redness when using LCI in the duodenal bulb.

## 4. Discussion

In this study, we conducted a retrospective study that focused on mucosal redness in the duodenal bulb as detected with LCI (the red component of the duodenal bulb with LCI was analyzed as a* values through objective evaluation) using EGD. We examined the association of mucosal redness with background factors and abdominal symptom-related QOL scores. We found that LCI enhanced mucosal redness in the duodenal bulb. Independent predictors of the red component (a*) of the duodenal bulb using LCI were a lower age, being male, a lower BMI, a lower EGAS, post-*H. pylori* eradication, and a lower constipation-related QOL score. This is the first study to objectively analyze mucosal redness in the duodenal bulb using LCI and to show a relationship with clinical background factors and abdominal symptoms. To date, we have not found a prior study that has investigated the relationship between duodenal mucosal color with IEE and abdominal symptoms.

Subjective evaluations of the mucosal color of the duodenal bulb were made using LCI. Approximately 21 (7.0%) out of 302 cases were in the redness (+) group. In this study, the participants were mainly hospitalized elderly patients with a relatively high *H. pylori* infection rate. However, the number of patients in the redness (+) group in LCI would be expected to increase if the average age was younger, such as when health screenings are performed. Several cases of partial or light redness were subjectively evaluated. For a more objective analysis, the L* a* b* color values of the mucosal color of the duodenal bulb were evaluated using LCI.

In recent years, with the development of IEE and image analysis, the evaluation of LCI for gastritis with L* a* b* color values has increased [30]. Sakae et al. objectively evaluated the color change of the gastric mucosa after *H. pylori* eradication [31]. In the corpus, the a* value with LCI and WLI decreased significantly after *H. pylori* eradication therapy. The color difference (Δ*E*) was significantly larger using LCI compared to WLI. In addition, a* values were found to be generally associated with neutrophil infiltration as determined by histopathology. From our previous study using L* a* b* color values [32,33], we reported that mucosa showing inflammation, such as in reflux esophagitis, became reddish with LCI. Therefore, we considered that a* values in LCI sensitively reflect mucosal inflammation. Taking into account the background of duodenal microinflammation and FD, we focused on the color tone of the redness of the duodenal bulb.

In this study, a* values using LCI were significantly higher than when using WLI in the redness (+) group but were significantly lower in the redness (−) group. This suggested that the redness tone was emphasized in the redness (+) group compared to the redness (−) group when using LCI. Furthermore, comparing the redness (+) and (−) groups, a* values were significantly higher in the redness (+) group for both WLI and LCI; however, no difference was observed in b* values with LCI. For WLI, not only the a* value but the b* value was also high in the redness (+) group, and the color tone looked more orange or brownish than red. For LCI, the b* value remained unchanged and the a* value was high so that it was highlighted in red. Therefore, even in cases where endoscopists cannot judge redness using WLI, using LCI seemed to allow for the assessment of redness by emphasizing the redness tone.

To investigate the clinical significance of duodenal red mucosa in LCI, correlation and multiple regression analyses between the red component (a*) of the duodenal bulb with LCI and clinical parameters were performed. A previous report investigated the correlation between mucosal color using L* a* b* color values with LCI and the vascular diameters or vascular angles of early esophageal cancer [34]. The vascular diameter of the intrapapillary capillary loops positively correlated with the b* color value. In this study, a lower age, BMI, EGAS, and constipation-related QOL score, plus post-*H. pylori* eradication, and being male were associated with the red component (a*) of the duodenal bulb when using LCI. In recent years, novel concepts in the relationship between the duodenum and FD, such as duodenal microbiota [35] or the presence of a duodenal barrier defect and immune activation [7], have arisen. Considering the relationship between microinflammation and symptoms such as FD, we initially hypothesized that duodenal redness in LCI may be associated with FD symptoms. With regard to the correlation between the red component of the duodenal bulb with LCI and various clinical parameters, upper abdominal pain–related QOL scores were significantly associated; however, Rho was low and excluded in our multiple regression analysis. Contrary to our hypothesis, our results highlighted that the observation of mucosal redness of the duodenal bulb using LCI is associated with a young population that showed less constipation and atrophic gastritis, and post-*H. pylori* eradication without FD symptoms.

A younger age, less atrophic gastritis, being male, and post-*H. pylori* eradication suggest that gastric acid may have an effect on duodenal redness in LCI. Ono et al. reported that spraying l-menthol sharpens the lavender color of intestinal metaplasia in the antrum when using LCI, emphasizing the color difference from the surrounding mucosa [36]. After spraying with l-menthol, the surrounding mucosa sometimes becomes reddish. This may be due to the direct chemical effect of l-menthol or the influence of acids since the pH of l-menthol is around 4.95. Thus, LCI is sensitive to the presence of l-menthol in the stomach and may also show redness in the duodenum in a hyperacid environment. Although PPI/PCAB non-users were excluded from this study in the multiple regression analysis, the many elderly participants and patients with open-type atrophic gastritis may have had lower gastric acid secretion. It is thought that a high BMI is related to a high level of gastric acid secretion; we found that a lower BMI was a risk factor for duodenal red mucosa in LCI. Although why lower BMI is associated with duodenal redness when using LCI is unclear, this may have been influenced by the fact that this study had more elderly participants and a higher proportion of participants with a low BMI than the general population. In the future, trials will be needed that include a younger population such as that found when conducting medical check-ups.

The influence of a history of alcohol use was also considered. Alcohol is known to cause upper gastrointestinal injury [37]. Excessive alcohol intake leads to increased gastric acid secretion and decreased gastric peristalsis [38]. A history of alcohol use was excluded from this study in the multiple regression analysis, which may have been influenced by its low prevalence of 6.5% in the study participants. In addition, the duodenal environment may be affected by bile. Our previous study used blue laser imaging (BLI) and revealed variables that affected the bile area in duodenal bulbs [39]. In a field of view, the proportion of bile area within the duodenal bulb with BLI was associated with cholecystectomy and the Bristol Stool Form Scale score. Because bile affects fecal characteristics and may affect duodenal redness in LCI, further investigations, such as analyzing bile volume in the duodenum or the monitoring of pH, are required.

Our study has several limitations. First, since the study was single-center, hospital based, and retrospective, a causal relationship between mucosal redness in the duodenum with LCI and clinical parameters could not be established. Second, subjective evaluations may have had observer bias. Evaluations were only undertaken in the duodenal bulb. The descending or deeper portion of the duodenum was not assessed because it was difficult to obtain reproducible subjective evaluations and objective analyses of mucosal color. Third, because the duodenum was not histologically evaluated, the pathological relationship with duodenal redness in LCI was not investigated. We were also unable to collect gastric juice or bile or analyze pH monitoring tests in the duodenum.

## 5. Conclusions

We found that 7.0% of the study participants showed redness in the duodenal bulb with LCI in a subjective evaluation. A positive relationship was observed between the red component (a*) of the duodenal bulb when using LCI and a lower age, BMI, EGAS, and constipation-related QOL score, as well as post-*H. pylori* eradication and being male in this hospital-based, cross-sectional study.

## Figures and Tables

**Table 1:** Clinical characteristics of the study participants (*n* = 302).

Characteristics	Value
Age in years, mean (SD, range)	70.9 (11.2, 27–92)
Sex (male: female)	120:182
BMI (SD)	22.8 (3.4)
Reflux esophagitis	None: 278, Present: 24
*H. pylori*	Negative: 186, Positive: 76, Post-eradication: 40
Atrophic gastritis	C-0: 95, C-1–3: 89, O-1–3: 118
PPI/PCAB use	Non-users: 191, Users: 111
Antiplatelet drug use	Non-users: 243, Users: 59
NSAID use	Non-users: 269, Users: 33
History of alcohol use	None: 283, Present: 19
History of tobacco use	None: 296, Present: 6

BMI, body mass index (kg/m^2^); *H. pylori*, *Helicobacter pylori*; NSAIDs, non-steroidal anti-inflammatory drugs; PPI, proton pump inhibitor; PCAB, potassium competitive acid blocker; SD, standard deviation.

**Table 2:** Abdominal symptom questionnaire scores of the study participants (*n* = 302).

Characteristics	Median	IQR	Range
Reflux-related QOL score	0	0–2	0–11
Upper abdominal pain-related QOL score	0	0–1	0–14
Fullness-related QOL score	0	0–2	0–12
Constipation-related QOL score	1	0–2	0–10
Diarrhea-related QOL score	0	0–1	0–15

IQR, interquartile range; QOL, quality of life.

**Table 3:** Objective analysis using L*, a*, and b* color values in the duodenal bulbs.

	L*, a* b* Values	WLI	LCI	*p*-Value
				WLI vs. LCI
All	L*	55.9 (50.1–61.3)	57.5 (51.7–63.1)	0.011
	a*	26.2 (22.2–32.0)	20.6 (13.9–27.5)	<0.001
	b*	35.0 (30.8–39.4)	31.3 (24.5–37.4)	<0.001
Redness (+)	L*	58.9 (52.6–64.5)	51.4 (46.2–56.8)	0.001
	a*	33.1 (30.3–37.2)	40.5 (37.5–41.9)	<0.001
	b*	36.6 (35.0–43.9)	31.2 (21.3–34.6)	<0.001
Redness (−)	L*	55.7 (49.9–61.1)	57.6 (52.0–63.2)	<0.001
	a*	25.7 (21.7–30.6)	20.2 (13.8–26.3)	<0.001
	b*	34.6 (30.2–39.1)	31.3 (25.4–37.7)	<0.001

Median (interquartile range); LCI, linked color imaging; WLI, white light imaging.

**Table 4:** Correlation between the red component (a*) of the duodenal bulb with LCI and various clinical parameters.

Clinical Parameters	Rho	*p*-Value
Age	−0.223	<0.001
Female	−0.069	0.232
BMI	−0.068	0.237
Reflux esophagitis	0.012	0.832
*H. pylori*-positive	0.002	0.969
*H. pylori*-eradicated	0.149	0.010
EGAS	−0.149	0.010
PPI/PCAB use	−0.138	0.017
Antiplatelet drug use	−0.087	0.133
NSAID use	−0.085	0.139
History of alcohol use	−0.072	0.215
History of tobacco use	−0.010	0.867
Reflux-related QOL score	0.011	0.845
Upper abdominal pain-related QOL score	0.118	0.041
Fullness-related QOL score	−0.061	0.289
Constipation-related QOL score	−0.082	0.155
Diarrhea-related QOL score	0.004	0.944

BMI, body mass index; EGAS, endoscopic gastric mucosal atrophy score; *H. pylori*, *Helicobacter pylori*; NSAIDs, non-steroidal anti-inflammatory drugs; PPI, proton pump inhibitor; PCAB, potassium competitive acid blocker; QOL, quality of life; Rho, Spearman’s correlation coefficient.

**Table 5:** Association between the red component (a*) of the duodenal bulb with LCI and other variables in the multiple regression analysis.

Variables	β	*p*-Value
Age	−0.154	0.009
Female	−0.129	0.024
BMI	−0.136	0.016
*H. pylori*-eradicated	0.137	0.015
EGAS	−0.149	0.013
Constipation-related QOL score	−0.122	0.027

BMI, body mass index; EGAS, endoscopic gastric mucosal atrophy score; *H. pylori*, *Helicobacter pylori*; QOL: quality of life; β, standardized partial regression coefficient.

## Data Availability

Data are contained within the article.
